# “Medical tourism will…obligate physicians to elevate their level so that they can compete”: a qualitative exploration of the anticipated impacts of inbound medical tourism on health human resources in Guatemala

**DOI:** 10.1186/s12960-019-0395-z

**Published:** 2019-07-12

**Authors:** Valorie A. Crooks, Ronald Labonté, Alejandro Ceron, Rory Johnston, Jeremy Snyder, Marcie Snyder

**Affiliations:** 1 Department of Geography, Simon Fraser University, 8888 University Drive, Burnaby, British Columbia V5A 1S6 Canada; 2 School of Epidemiology and Public Health, University of Ottawa, Alta Vista Campus, 600 Peter Morand Crescent, Ottawa, Ontario K1G 5Z3 Canada; 3 Department of Anthropology, University of Denver, 2000 E. Asbury Avenue, Denver, CO 80208 USA; 4 Faculty of Health Sciences, Simon Fraser University, 8888 University Drive, Burnaby, British Columbia V5A 1S6 Canada; 5 Meating Ground Consulting, Collingwood, Ontario, Canada

**Keywords:** Medical tourism, Health equity, Guatemala, Health human resources, Latin American and Caribbean region

## Abstract

**Background:**

Medical tourism, which involves cross-border travel to access private, non-emergency medical interventions, is growing in many Latin American Caribbean countries. The commodification and export of private health services is often promoted due to perceived economic benefits. Research indicates growing concern for health inequities caused by medical tourism, which includes its impact on health human resources, yet little research addresses the impacts of medical tourism on health human resources in destination countries and the subsequent impacts for health equity. To address this gap, we use a case study approach to identify anticipated impacts of medical tourism sector development on health human resources and the implications for health equity in Guatemala.

**Methods:**

After undertaking an extensive review of media and policy discussions in Guatemala’s medical tourism sector and site visits observing first-hand the complex dynamics of this sector, in-depth key informant interviews were conducted with 50 purposefully selected medical tourism stakeholders in representing five key sectors: public health care, private health care, health human resources, civil society, and government. Participants were identified using multiple recruitment methods. Interviews were transcribed in English. Transcripts were reviewed to identify emerging themes and were coded accordingly. The coding scheme was tested for integrity and thematic analysis ensued. Data were analysed thematically.

**Results:**

Findings revealed five areas of concern that relate to Guatemala’s nascent medical tourism sector development and its anticipated impacts on health human resources: the impetus to meet international training and practice standards; opportunities and demand for English language training and competency among health workers; health worker migration from public to private sector; job creation and labour market augmentation as a result of medical tourism; and the demand for specialist care. These thematic areas present opportunities and challenges for health workers and the health care system.

**Conclusion:**

From a health equity perspective, the results question the responsibility of Guatemala’s medical education system for supporting an enhanced medical tourism sector, particularly with an increasing focus on the demand for private clinics, specific specialities, English-language training, and international standards. Further, significant health inequalities and barriers to care for Indigenous populations are unlikely to benefit from the impacts identified from participants, as is true for rural-urban and public-private health human resource migration.

## Background

When individuals intentionally travel across international borders to access private, non-emergency medical interventions that are unavailable, delayed, unsafe, relatively inaccessible, or too costly in their country of residence they are taking part in a practice that has come to be popularly known as medical tourism [1, 2]. At times, individuals are driven to consider international health care options due to inequities embedded within their home systems that prevent timely, local, or affordable access to care [2]. The intentional nature of this practice differentiates medical tourism from the care that is provided to ill and injured vacationers, while the private nature of this arrangement differentiates medical tourism from cross-border care for international patients that is coordinated and paid for by governments or their agencies [2]. In other words, this practice takes place outside established cross-border care agreements between countries. Consequently, it does not require physician referral and care is typically paid for out-of-pocket by the medical tourist or their family [3–6].

Increased health care privatization coupled with trade liberalization has created a market atmosphere for Global South countries in particular to commodify and export their private health services to international medical tourists as a form of offshoring [7]. As a result, the economic significance of medical tourism in the global marketplace is growing [8]. Indeed, countries around the world are increasingly competing for medical tourists, and the number of Global South destination countries serving as destinations is growing, largely due to the perceived economic benefits of this sector [9, 10]. Many Latin American and Caribbean (LAC) region countries, for example, are seeking to get into or expand this sector due to perceived benefits, such as increased foreign revenue [11]. There has been increased emphasis on promoting medical tourism to the LAC region via government initiatives and other strategic marketing platforms that often target patients travelling from the United States (US) and Canada or from elsewhere within the LAC region [1, 12]. Varied procedures are offered across the hospitals and clinics seeking to attract medical tourists, including cardiac, orthopaedic, cosmetic, and dental surgeries [13, 14].

Concern for the ways in which the growth and development of medical tourism can create or exacerbate health inequities in destination countries is growing, the implications of which can be complex [15]. Generally speaking, health inequities are unfair differences in health status within or between populations or individuals that are generated by structures and systems that disadvantage some groups over others, including in relation to access to health services or opportunities for achieving health (e.g. income, safe housing), yet that are avoidable [16]. Health (in)equity is associated with medical tourism in destination countries in that it may impact how fairly health care access and distribution is achieved at the local level based on international patients’ use of these same (often constrained) resources [17]. The challenges associated with balancing local and foreign interests can give rise to health equity debates around medical tourism in the LAC region [18]. For example, factors that may result in gains for one group, such as the ease of access to care for international patients, may harm another, such as health workers in countries with health human resource shortages being employed in clinics treating international patients rather than at understaffed public hospitals (e.g. [19]). As another example, research in the LAC region has demonstrated that while health care providers and policymakers often believe that development of a national medical tourism sector can be an opportunity for economic growth and enhancing equitable access to improved care for local patients, high-end or specialized services targeting international patients may actually be too costly for most local patients to access, thereby increasing health disparities at the local level [20].

There has been open concern for how medical tourism can and does impact health human resources in destination countries, and its implications for health equity [21–23]. For example, the higher rates of pay sometimes offered to health workers employed in clinics targeting international medical tourists may serve to retain skilled health workers [8, 24]. Conversely, the redistribution of health workers from public to private sector, or from rural to urban clinics, exacerbates inequities in the distribution of health human resources [1, 20]. Research by Snyder et al. [24] in the LAC region country of Barbados shows that articulating the harms and/or benefits of these impacts is very challenging as local medical tourism stakeholders from different sectors can understand the same impacts in different ways. For example, interviews with medical tourism stakeholders in Barbados showed that while stakeholders involved in the tourism and economic development sectors may focus on the local benefits of more health worker jobs brought about by medical tourism, representatives of the health and civil society sectors may see this same impact as signaling harms for the public sector that is being depleted of workers who migrate to work at high-paying specialist clinics. Despite some important existing research that has explored how medical tourism and health human resources sectors intersect and the implications of these intersections for destinations (e.g. [8, 11, 24, 25]), syntheses of the literature examining the health equity impacts of medical tourism for destination countries consistently identify the need for more research on such intersections (e.g. [2, 15, 21, 26, 27].

The purpose of this paper is to identify the anticipated or forward-looking impacts of the developing medical tourism sector on health human resources in Guatemala, a Spanish-speaking country in the LAC region. To do this, we report on the findings of 50 key informant interviews conducted with local medical tourism sector stakeholders across four groups. Given the nascent stage of Guatemala’s medical tourism sector, this analysis provides important early, forward-looking insight into anticipated negative health equity impacts that have the potential to be offset before ever being realized through precautionary or preventative policy-making. This case study also contributes novel empirical insights into what is an emerging evidence base of medical tourism’s impacts on health human resources in emerging LAC region destinations and, ultimately, on health equity in countries developing this sector. The findings and their implications may hold transferability for nearby LAC region countries as diverse as Belize, where local residents have expressed concern over the potential for medical tourism to result in health worker shortages at clinics [28]; Cayman Islands, where international health workers from India are staffing the country’s main medical tourism facility and in doing so are placing strain on its limited housing rental market and public infrastructure [29, 30]; Jamaica, where it has been suggested that if medical tourism development places strain on specialist health worker resources, the political will to drive this sector forward will be lessened [31]; the Bahamas and Turks and Caicos, where medical tourists can travel with surgeons from their home country to clinics in these countries for experimental treatments, which poses regulatory challenges regarding physician licensing and insurance [12, 32]; and Costa Rica, where health workers have championed having facilities obtain international accreditations as a way of easing international patients’ concerns regarding care quality and ultimately growing the potential of the medical tourism sector [33]. This is not to suggest that the findings cannot also have relevance to less proximal LAC countries seeking engagement in this sector, such as Brazil [34] and Argentina [35], or those with more established medical tourism sectors, such as Mexico and Cuba [36].

## Context

Guatemala is a Central American country that is just over 100,000 km^2^ in size and is home to an estimated 15.5 million people. An estimated 52.5% of the population is urban, with almost half the total population identifying as Indigenous [37]. A high proportion of the population is quite young, with 34.5% under the age of 15 and 95.46% under the age of 65. The main language spoken is Spanish, although 23 Amerindian languages are also recognized here [38].

Guatemala is a lower-middle income country in the LAC region, with a notably high level of income inequality. Typically, Guatemala devotes a low proportion of its GDP to social spending, making it one of the lowest in the region. Indeed, because of this, the country endures significant challenges to its health care system, along with a population that experiences systemic socio-economic and health-related inequities. According to the UN’s Human Development Index (HDI)—which measures life expectancy, access to knowledge, and standard of living—Guatemala ranks 125 out of 169 countries, making it the second lowest ranking country in the LAC region [39]. More than half the population lives below the national poverty line, and poverty among Indigenous groups is very high at 79% [38]. Guatemala has the highest infant mortality rates and lowest life expectancy of any Central American country. Malnutrition among children is higher than any other Latin American country [40, 41].

In recent years, Guatemala has made limited progress in terms of improving its health system and in providing equitable access to care. In 2014, 6.2% of the GDP was spent on health expenditures, and the country offered only 0.6 hospital beds/1000 people and 0.9 physicians/1000 people in 2009 [38]. The health sector is composed of a network of public, private non-profit, and private for-profit institutions, however, with an under-resourced public health care system, health care coverage is not consistent, nor is it comprehensive. Most Guatemalans rely on charitable or public care. The Ministry of Public Health and Social Welfare runs about 1,304 hospitals and health facilities [41], with most hospitals located in Guatemala City. The concentration of health human resources mainly in urban areas, namely Guatemala City, leaves rural areas under-served. The highest rate of health service expansion in recent years has been in the private sector [41]. Health service access is particularly challenging for Indigenous communities who experience transportation, language, discrimination, and cultural barriers to care [40].

Guatemala has a small yet growing medical tourism sector catering to international patients, including members of its diaspora community living elsewhere in the LAC region as well as patients from the United States of America and Canada. To make private health care facilities in Guatemala City and Antigua, Guatemala—the two main tourist centres in the country—attractive to international patients, individual clinics and government strategies promote cost competitive services, and some larger clinics are moving to improve their attractiveness to international patients by seeking Joint Commission International accreditation [41]. The planned development of this sector relies heavily on utilizing existing health care infrastructure, primarily unused capacity in private hospitals and clinics, rather than building new facilities to accommodate medical tourists. Initiatives to develop this offshoring sector, including the creation of strategic working initiatives, are primarily led by Guatemala’s Tourism Commission for Health and Wellness. To attract US patients to Guatemala and in an effort to better organize this new sector, private hospitals, hotels, airlines, individuals, and private companies have also recently created a formal network (the Guatemalan Exporters Association) to facilitate exporting health services [11, 41, 42].

## Methods

The purpose of this exploratory qualitative study was to identify the anticipated impacts of the growth and development of the medical tourism industry in the LAC region on health (in)equities. To do so, we conducted in-depth interviews with key informants that examined positive and negative health system and policy changes across five domains: (1) public health care, (2) private health care, (3) health human resources, (4) investment, and (5) domestic government involvement. Within these five domains, we considered impacts that have already occurred, as well as anticipated impacts. Elsewhere, we have published analyses of these interviews that have examined the potential roles for the Guatemalan government to regulate domestic medical tourism development [43] and identified the ways in which existing health system inequities are providing opportunities to drive medical tourism forward in Guatemala [44]. This research was guided by the comparative case study methodology, which informs nuanced understandings of a particular phenomenon, taking into consideration the context in which it occurs, and by drawing on multiple sources of data and information [45]. We have also incorporated these key informant interviews into two comparative analyses: one that compares Guatemalan and Barbadian stakeholders’ understandings of the potential to draw medical tourists to their countries [11]; and another that comparatively identifies factors that are driving and inhibiting medical tourism development in Guatemala, Barbados, and Mexico [46]. The current analysis is the only one emerging from the comparative case study to explore the forward-looking impacts of medical tourism sector development on health human resources in any of our LAC destination countries of focus, which was a rich part of discussion among Guatemalan key informants.

To assist with understanding the context of this case study, we first conducted an extensive review of media and policy discussions about Guatemala’s medical tourism sector and compiled a background document summarizing key health and health service indicators for the country [41]. Following this, two of the lead investigators visited Guatemala to partner with the Guatemalan research team and tour public and private health care facilities. Another purpose of this developmental site visit was to informally speak with policy officials, representatives from the country’s two committees working to promote and develop medical tourism, and health workers, to observe and understand first-hand the complex dynamics that surround developing the medical tourism sector. Our next step was to conduct key informant interviews to identify the anticipated health equity impacts of the country’s planned development of a medical tourism sector.

### Recruitment

Following ethics approval, we purposefully recruited 50 people to participate in semi-structured face-to-face interviews. Purposeful recruitment is particularly valuable for identifying participants with specific knowledge or insights into the area of study, and who are able to provide information-rich understandings of any related concerns [47]. Potential participants were identified by locating names in our review of media and policy documents, through the networks of our local non-governmental organization (NGO) collaborators, and when existing participants shared study information with others in their networks. We sought key informants among five sectors: (1) civil society, which included NGOs, local chapters of international organizations, community groups, and the media; (2) health human resources representatives, comprising health workers, medical education professionals, and health worker union representatives; (3) government representatives, including government ministry staff; (4) public hospital and health care administrators, tourism officials, and investment sector representatives; and (5) private health care or tourism representatives, consisting of tourism consultants, owners of private health care clinics, private investment experts, and investors. We spoke with five representatives from civil society organizations, 15 representing health human resources agencies or departments, 15 representing public health care, and 15 representing private health care. To protect participants’ anonymity, we do not provide a more detailed breakdown regarding their employment contexts or professional experience.

Most potential participants were initially contacted via emails that explained the study, the importance of their perspective, and the interview details. In some cases, potential participants were approached in-person or by phone if their position best suited being contacted this way. Those who received an invitation to participate in an interview were asked to reply via phone or email to express their interest in contributing to the study. After doing so, an interview was scheduled by one of the two Guatemala-based interviewers at an agreed upon time and location.

### Data collection

In-depth interviews were conducted in Spanish, in Antigua Guatemala and Guatemala City, over a 7-month period by a research associate and assistant hired and trained by our partner NGO. Interviewees were informed of their rights as a participant in a research study and gave verbal consent to participate in the study, which was recorded on a form that the interviewer signed and dated. Interviews ranged from 45 to 90 min in length. Most interviews were conducted one-on-one, although in one case a small group interview was conducted based on participant preference.

Interview questions were developed based on prompts emerging from (1) our scoping review of the academic, grey, and media literature on the health equity impacts of medical tourism [2]; (2) our detailed background report that explored the context for the development of medical tourism in Guatemala [41]; and (3) a review of insights gathered during our earlier pilot study exploring the health equity impacts of medical tourism in the LAC country of Barbados [24]. The interview guide was organized around a set of common questions that were asked of all participants, as well as a set of questions tailored to participants’ area(s) of expertise. General questions included inquiries into common health system challenges and opportunities as well as knowledge of local health systems and medical tourism. Most of the questions focused on the five domains of health equity interest. With regard to health human resources, participants were asked about the anticipated or forward-looking impacts on medical education, job availability, specialist demand, public-to-private movement of workers, patient interaction, training to treat international patients, and remuneration, among other factors. The guide was semi-structured and so participants were able to discuss topics not covered in the questions that they felt were important. The interview guide was developed in English with input from all members of the international team. It was then translated and back-translated by the research associate and assistant based in Guatemala to ensure accuracy.

### Data analysis

Interviews were digitally recorded and simultaneously translated into English and transcribed. A select number of transcripts were then independently reviewed by the lead investigators to identify initial themes in preparation for thematic analysis. Thematic analysis involves categorizing data into themes, based on overarching patterns in the dataset, and contrasting these themes to the study objectives and existing literature as a way to gain new insights [48]. A face-to-face meeting was held among investigators following an independent transcript review in order to identify topics for thematic analysis and co-develop a coding scheme. It was through this process that the focus of the current analysis emerged as being significant. Following this, one lead investigator drafted a proposed full coding scheme that inductively and deductively captured the analytic directions identified in the team meeting. Feedback was next solicited to gain consensus on the scheme. All transcripts were then imported into NVivo, a qualitative data management program that facilitates collaborative analysis, in preparation for coding. To test the integrity of the coding scheme, five transcripts were independently coded by an investigator, after which the lead investigator reviewed the coding to troubleshoot, eliminate redundancy, and confirm the scope of each code. The full dataset was next coded using the revised scheme by a single investigator to ensure consistency and methodological rigour.

After coding was complete, the investigator who oversaw coding and the lead investigator together extracted all coded data related to health human resources in the Guatemala case and reviewed the extracts to identify emergent themes. Triangulated review of these extracts led to the identification of five themes emerged regarding the anticipated impacts of an expanded medical tourism sector on health human resources in Guatemala. Coding extracts for each theme were shared with the full team in order to confirm interpretation and the scope and scale of each theme. Following this, and in keeping with the thematic analysis process [48], the emerging findings were contrasted against the existing literature to refine the analysis. This collaborative, triangulated, iterative process culminated in five agreed upon key themes that are discussed in the section that follows.

## Results

Findings from key informant interviews with 50 participants across four stakeholder groups revealed five key thematic areas of focus that relate to medical tourism development and its anticipated impacts on health human resources in Guatemala. These issues include stakeholder perceptions of the impetus, or push, to meet international standards and accreditations in health care training and practice; opportunities and demand for English language training for health workers; the migration or movement of health workers from the public to private sectors and within the private sector; the creation of new jobs and augmentation of labour market competition as a result of medical tourism; and the demand for more specialist care. Participants indicated that these anticipated impacts, or areas of concern, included both opportunities and challenges for the health professions as well as for Guatemala’s health system, which we explore in greater detail with regard to implications for health equity in the discussion section. While the five themes are presented separately below, we acknowledge interrelationships, some of which we explore in the discussion section that follows.

### Impetus to meet international standards in training and practice

The most commonly discussed anticipated impact of an expanded inbound medical tourism sector on health human resources in Guatemala pertained to the impetus it could create for healthcare providers to meet international standards, or to become certified by international regulating bodies. It was thought that this may ensure that the health services provided to medical tourists were on par with those of other competing medical tourism destinations in terms of quality and health outcomes. As one participant explained, “medical tourism will … obligate [Guatemalan] physicians to elevate their level so that they can compete with the rest of the world.” Another participant similarly noted that “far from having a negative impact, it [medical tourism] would be something really positive, because the professionalization of the staff in the health system gets better.” From this perspective, an expanded medical tourism sector could motivate Guatemala’s healthcare providers to upgrade their skills and practice which, in turn, could benefit local patients as well.

The growth of medical tourism in the LAC region and proposed expansion in Guatemala could also incentivize hospitals and clinics seeking to treat international patients to obtain internationally-recognized facility accreditation (e.g. Joint Commission International). A private sector provider interested in treating more international patients mentioned that within their clinic “when we are preforming a surgery, we meet all the standards and procedures indicated [by the international accreditor]. And we also want all the personnel that work within the equipment to have their certification.” Many participants believed that international patients seek out clinics that have international accreditations, and that such accreditations would therefore be necessary for Guatemala to position its medical tourism sector regionally and internationally. There was also awareness that such accreditations have direct implications for health workers as they often involve the development of staffing protocols and role profiles. One participant noted that the push toward international accreditation already exists within the university sector, including institutions training health workers. It was explained that as a result, “twenty years ago, only students’ [skills] were evaluated. Nowadays … teachers, facilities… everything’s being evaluated” for competency against various standards. The push towards this accreditation of hospitals and clinics to gain or expand entry into the medical tourism sector, however, also had participants raising questions about the impact that seeking accreditation might have on the day-to-day functioning of these facilities and their health workers (e.g. time-consuming routine evaluations, costly ongoing training).

### Opportunities and demand for English-language training and competency

Most participants agreed that attracting patients from nearby English-speaking countries (the United States of America and Canada) would be a priority for Guatemala’s medical tourism sector. It is therefore not surprising that the need for greater English-language training among health workers was commonly discussed by participants as an anticipated impact of growth in the medical tourism sector. For example, a private health facility supervisor expressed that “most of us who work in medical tourism need to communicate in English. I’m not 100% proficient, but I can make my patients understand me. However, my staff doesn’t have that ability.” This interviewee went on to positively suggest that “English should become a second language in the country. This is the international language. European and North American patients communicate in English,” underscoring the anticipated need for English language training among local healthcare workers. It was also acknowledged that English is the preferred language for some second and third generation Guatemalan expats living abroad and that providing medical services in English may encourage them to seek treatment in Guatemala while visiting family. It was frequently suggested that language training should be provided in medical education programming rather than having clinics and hospitals in the medical tourism sector paying privately for courses for their staff.

It was thought that a lack of English language competency could create barriers between health workers and international patients in an expanded medical tourism sector: “what exists is the language barrier…due to the education we have here…language is the biggest barrier.” As another participant explained:

Definitely, more emphasis on learning English should be given by medical professionals … Because it already is hard for a patient to take the decision to go to a country they have never been to, with a physician they have never seen, in a country where their native language isn’t spoken. Having a fluent conversation with a patient will in some way lower the stress of all the involved parties.

Being cared for by a group of medical personnel that speak fluent English could potentially make the health care experience less intimidating for non-Spanish speaking medical tourists, and could also improve the reputation of the country as a suitable medical tourism destination.

### Migration and movement of health workers

Several participants suggested that the practice of medical tourism in Guatemala could work to drive health workers from the public to the private sector. This discussion most commonly centred around physician migration, as opposed to the movement of nursing staff from one sector to the other. For example, one participant anticipated that those most likely to migrate from public to private practice “would be the clinical physicians”, and went on to critically explain that even without medical tourism:

…there is a migration in the positions of the public sector which are the ones with the lower salaries, towards the private sector or an NGO or a corporation agency…the same thing could happen with medical tourism. If it seems that there are more resources or a major capacity to generate income there, then people would go in that direction

As such, it was noted that expanded medical tourism sector in Guatemala may exacerbate an existing trend regarding physician migration into the private sector that is harming public health care in the country. It was explained that physicians who are “well prepared”, which includes some degree of English-language fluency and the ability to offer procedures and treatments sought by medical tourists, will be able to take advantage of moving into the private sector.

The potential for obtaining higher salaries through treating international patients in private clinics was thought to be a motivator for the anticipated public-to-private migration, or for physicians who work in both sectors to increase their private sector hours and decrease their public sector ones. As one participant explained: “Currently there are private hospitals, that due to the fact that they have specialized treatments and with better quality, they have the ability to pay their personnel better. People would prefer to work in those places.” The potential to work in clinics and hospitals with more specialized or advanced technology was also cited as a driving factor for moving into private practice. Participants expressed that the perception of working in a resource-rich clinical setting targeting medical tourists is likely to serve as one of the key factors driving internal public-private migration.

### New jobs and labour market competition

Participants widely anticipated that an expanded medical tourism sector would bring foreign investment into Guatemala, and in doing so new employment opportunities would open up in hospitals and clinics treating international patients. It was explained that medical tourism “gives the communities the opportunity to take part in this business…it opens up [opportunities] to doctors that will take advantage of this offer.” Spurred by these opportunities, “physician[s] will begin to see more and more patients, and will need to hire nurses and a bigger clinic to treat these patients. He [or she] will need a place to accommodate the people that come. This will generate more hotel use and hospital use.” As this comment suggests, several participants anticipated that medical tourism would have a positive impact on employment extending beyond hospitals and clinics and into the country’s hospitality and tourism sector.

In addition to opening up new employment opportunities, some participants also acknowledged that an expanded medical tourism sector may introduce new forms of labour market competition. Specifically, clinics and hospitals could begin to compete against one another for what they consider to be the most desirable workers—those who speak English fluently and/or practice in areas that are in demand by medical tourists.

It generates employment. It makes local actors compete, and that makes the already existing companies more productive and it makes them increase their quality levels. It also creates [competition] in the labour market. For example, if nurses are needed, only the best would get hired…at the top of their game…

Not only did participants comment about the potential for public-to-private migration of health workers, as discussed in the previous sub-section, but also for competition to exist within the private sector for sought after employees. As the last quote suggests, some viewed this competition as potentially leading to better quality care delivery for medical tourists.

### Specialist demand

An anticipated impact raised by only a few participants pertained to the potential for an expanded medical tourism sector to create new demand for specialists who perform procedures that are in high demand by medical tourists (e.g. cosmetic and plastic surgery, dental surgery and prosthodontic specialists). This anticipated demand, however, would extend beyond physicians to include specialists in “nurse personnel, technical personnel, and maybe [administrative] professional at a technical level.” It was explained that this increase in demand would not always result in a need for new health workers as there were some private specialists working in fields where “there is not [a] big market” in Guatemala at present. Some participants thus suggested that medical tourism may assist with using excess capacity rather than incentivizing new supply in some segments of the specialist health care market.

## Discussion

In the previous section, we presented the thematic findings of 50 interviews conducted with key informants in Guatemala that explored the realized and anticipated impacts of an expanded medical tourism sector on health (in)equity, and in this case health human resources. Five impacts of an expanded medical tourism sector on health workers emerged across this diverse participant group: (1) a push toward meeting international standards in training and practice; (2) greater demand for English-language training and competency; (3) increased migration from the public to private sector; (4) new employment opportunities and labour market competition; and (5) greater demand in some specialty practice. Many of these anticipated impacts are consistent with the findings of other studies in, and reports from, the LAC region. For example, our own research in Barbados has shown that a forward-looking hope is for the push for international accreditation to increase local standards of practice [24], while other research has documented forward-looking concern that new employment opportunities in private medical tourism clinics in El Salvador, Honduras, Guatemala, and Mexico could greatly negatively affect public health care [49]. The continued emergence of these anticipated impacts in the Guatemalan context offers further evidence that can be used to push governments and other regulatory bodies in destination countries to instil measures to prevent them from being realized or harming health equity [43]. Some participants viewed these impacts as positive, seeing medical tourism as having net benefits for Guatemala and its health workers, while others viewed these same ones as negative, viewing this practice as being harmful in part because it shifts health system priorities. This is also consistent with the wider medical tourism literature that has shown that stakeholders’ positioning plays a significant role in how they conceptualize the local impacts of the transnational practice of medical tourism [21, 46]. While acknowledging this dichotomy, here we consider the implications of these impacts for health (in)equity in Guatemala after first considering some of the interrelationships between the themes identified in this analysis.

We noted at the outset of the findings section that there are interrelationships between the five themes identified by our analysis. For example, participants anticipated greater demand for and interest in English-language training among health workers in the medical tourism sector. Other studies have suggested that English is the dominant language of the global medical tourism industry and that this reality heightens the importance of English competency among those treating medical tourists, including health care administrators and patient coordinators who interact routinely with international patients and the friends and family who accompany them abroad [46, 50]. This finding draws direct links to the themes surrounding the public-to-private migration of health workers and the opening of new employment positions. As suggested by the findings, these impacts are most likely to be felt among sectors and places where health workers with English-language skills commonly practice along with those that have specialized training or competency in treating international patients. The anticipated demand for specific specialities, English-language training, and use of international standards in training opportunities are also findings that are interrelated as they all have direct implications for Guatemala’s medical education system that is responsible for training health workers. Some participants expressly noted that an expanded medical tourism sector may necessitate changes in the medical education system. Though not commented on by our participants, the creation of specialized medical tourism-focused certificates and training programs at educational institutions have been documented elsewhere [9, 51]. Instead, several of the private hospital and clinic owners as well as medical tourism promotional committee members we spoke with during our facility tours in Guatemala suggested a more nuanced approach. It would involve, for example, English-language classes and international certifications becoming part of existing medical education programs rather than something that health workers pay for directly or private clinics seeking to treat international patients arranging for their staff via independent certifiers.

Many of the anticipated impacts of an expanded medical tourism sector in Guatemala on health human resources identified in this analysis have direct or indirect implications for medical education and training, which has the potential for being a meaningful focus of preventative or precautionary action to avoid exacerbating health inequities. These impacts include the anticipated push for English language competency among health workers, the likely need for health workers to meet international standards and assist with facilities obtaining international accreditations, and the anticipated overall need for educational institutions to train workers in areas of demand. Some of these direct and indirect implications will be realized pre-graduation, during the period in which future health workers are in the process of obtaining an education, while others will occur once on-the-job. From a health equity perspective, an important question emerges: what responsibility does or should Guatemala’s medical education system have for supporting an enhanced medical tourism sector? In Guatemala, there are both public and private medical education institutions [41]. It is widely acknowledged that medical schools have a social responsibility to serve the communities in which they are based as a way of enhancing health equity, which includes a return on investment for the public training of health workers through their practice in public systems [52, 53]. This even holds true for private medical education institutions because they typically benefit from some degree of public investment [54]. The anticipated impacts of an expanded medical tourism sector in Guatemala shift the focus of some aspects of medical education away from its social responsibility and thus may threaten health equity in doing so. From a health equity vantage, moving the costs of English-language training from providers to the education system represents a public subsidy for what would be a private medical practice. Although the public costs may be small, they nonetheless represent an allocation away from underfunded public health facilities into private, for-profit medical care inaccessible to most Guatemalans.

We noted in the introduction that there is a high level of income inequality in Guatemala and that the Indigenous population is particularly impoverished and experiences significant barriers in accessing health care, including language barriers. Spanish is the primary language of health care delivery, yet many Indigenous persons who speak one or more of the numerous Indigenous languages are unable to speak Spanish [55]. Although likely an artefact of our interview schedule (which probed only on the issue of medical tourism), the preponderance of concern for English-language training by participants and notable silence on issues of intercultural competencies is striking. Applying a health equity lens, improving language skills for Guatemalan health workers should prioritize improving the health system’s ability to serve its underserved Indigenous population over treating high-paying English-speaking international patients as a form of preventative action. The same health equity critique applies to participants’ comments regarding the potential for health workers to migrate from rural centres to urban ones, or from the public sector to the private one to treat high-paying international patients in medical tourism clinics. These anticipated impacts of an enhanced medical tourism sector on health human resources do nothing to address the rural-urban or public-private inequalities in health service delivery that drive important aspects of health inequity in Guatemala [2, 21].

It was explained earlier that there are low levels of government spending on public resources in Guatemala when compared to other countries in the LAC region, with much of the increased or enhanced capacity in the country’s health system coming via the private sector [40]. Ambitions regarding medical tourism sector development in the country rest largely with utilizing slack capacity in the private sector [41], a not uncommon finding in other lower and middle-income countries developing the industry [46]. Literature exploring the health equity impacts of medical tourism often suggests that such a strategy can have spill-over benefits for the public health care system through the redistribution of profits (through tax/transfer initiatives of the government) or enhanced access to medical technologies that can be shared between these sectors [2]. This literature is typically speculative in nature, and examples of such benefits emerging in medical tourism destinations are rarely evidence-based. In participants’ discussions of the anticipated impacts of medical tourism sector development on health human resources in Guatemala, there was a heavy focus on health worker gains and potentially positive impacts for the private system through treating international patients, whereas similar discussion of the public system and the benefits it may receive was quite absent. This is highly concerning given that investment in or growth of public health care is widely acknowledged to be the best way to address health inequalities and enhance health equity, particularly through creating robust primary health care [56–58]. Guatemala lacks a functioning and effective primary health care system [38, 54], and so it is a clear health equity-related concern that so many of the anticipated health system gains brought about by medical tourism are centred around the private system.

## Conclusion

Many of the issues raised in this analysis of 50 key informant stakeholder interviews from Guatemala regarding the anticipated impacts of medical tourism development on health human resources have some traction with the broader literature on health worker migration, which often refer to medical tourism as being a way to retain, or to attract the return of, émigré health professionals, notably physicians. Some of our own work in this area, however, shows little evidence of this occurring [59, 60]. Even if medical tourism does benefit health workers, it is more likely to personally benefit those “at the top of their game” (as one participant put it) than those treating poorer population groups in destination countries promoting medical tourism. This outcome appears rather starkly to be the case with Guatemala, where the findings of the current analysis and their implications suggest that a health system focus on enhancing care for English-speaking international patients that holds significant anticipated impacts for health human resources, for example, does little to address the inequities in access facing its indigenous populations or reversing the rural-to-urban brain drain of its health workforce. Much of this system enhancement will require support from the medical education sector, which we identified as a potential focus for preventative policy-making.

Health workers in Guatemala, and likely many other LAC region countries that are developing medical tourism sectors, who are “at the top of their game” are likely going to continue to benefit the most from any impacts brought forth by medical tourism as long as governments and regulators either lack capacities for, or interests in, regulation in the sector. As we have pointed out elsewhere, the regulatory potential for medical tourism in Guatemala is limited due to a lack of mechanisms to provide oversight, enforce necessary (policy) change, and ensure an equitable distribution of benefits [43] Having such benefits advantage only certain health workers is not likely to result in enhanced health equity in Guatemala or elsewhere, nor greater access to care for the most impoverished and underserved citizens. Such health equity outcomes are also not captured by aggregate welfare measures, which are often used to defend increasing international openness in “health mobilities”—whether of health workers (international migration), patients (medical tourism), or foreign investment (promoting privatized services).

## Data Availability

The datasets analysed during the current study are not publicly available due to privacy concerns, but anonymized versions may be made available by the corresponding author upon reasonable request.

## Abbreviations

HDI: Human Development Index
LAC: Latin American and Caribbean
NGO: Non-governmental organization
US: United States
